# Increased gut permeability and bacterial translocation are associated with fibromyalgia and myalgic encephalomyelitis/chronic fatigue syndrome: implications for disease-related biomarker discovery

**DOI:** 10.3389/fimmu.2023.1253121

**Published:** 2023-09-07

**Authors:** Franz Martín, Manuel Blanco-Suárez, Paola Zambrano, Oscar Cáceres, Miriam Almirall, José Alegre-Martín, Beatriz Lobo, Ana Maria González-Castro, Javier Santos, Joan Carles Domingo, Joanna Jurek, Jesús Castro-Marrero

**Affiliations:** ^1^ Andalusian Centre of Molecular Biology and Regenerative Medicine (CABIMER), University Pablo Olavide, University of Seville, Seville, Spain; ^2^ Biomedical Research Network on Diabetes and Related Metabolic Diseases (CIBERDEM), Instituto de Salud Carlos III, Madrid, Spain; ^3^ Central Sensitivity Unit (SHC Medical), Hospital Viamed Santa Ángela de la Cruz, Seville, Spain; ^4^ Division of Rheumatology, Vall d’Hebron University Hospital, Universitat Autònoma de Barcelona, Barcelona, Spain; ^5^ Rheumatology Research Group, Myalgic Encephalomyelitis/Chronic Fatigue Syndrome (ME/CFS) Research Unit, Vall d´Hebron Research Institute, Universitat Autònoma de Barcelona, Barcelona, Spain; ^6^ Laboratory of Neuro-Immuno-Gastroenterology, Digestive System Research Unit, Vall d’Hebron Research Institute, Vall d’Hebron University Hospital, Universitat Autònoma de Barcelona, Barcelona, Spain; ^7^ Centro de Investigación Biomédica en Red de Enfermedades Hepáticas y Digestivas (CIBERehd), Instituto de Salud Carlos III, Madrid, Spain; ^8^ Department of Biochemistry and Molecular Biomedicine, Faculty of Biology, University of Barcelona, Barcelona, Spain

**Keywords:** anti-beta-lactoglobulin, chronic fatigue syndrome, fibromyalgia, intestinal permeability, myalgic encephalomyelitis, zonulin

## Abstract

**Background:**

There is growing evidence of the significance of gastrointestinal complaints in the impairment of the intestinal mucosal barrier function and inflammation in fibromyalgia (FM) and in myalgic encephalomyelitis/chronic fatigue syndrome (ME/CFS). However, data on intestinal permeability and gut barrier dysfunction in FM and ME/CFS are still limited with conflicting results. This study aimed to assess circulating biomarkers potentially related to intestinal barrier dysfunction and bacterial translocation and their association with self-reported symptoms in these conditions.

**Methods:**

A pilot multicenter, cross-sectional cohort study with consecutive enrolment of 22 patients with FM, 30 with ME/CFS and 26 matched healthy controls. Plasma levels of anti-beta-lactoglobulin antibodies (IgG anti-β-LGB), zonulin-1 (ZO-1), lipopolysaccharides (LPS), soluble CD14 (sCD14) and interleukin-1-beta (IL-1β) were assayed using ELISA. Demographic and clinical characteristics of the participants were recorded using validated self-reported outcome measures. The diagnostic accuracy of each biomarker was assessed using the receiver operating characteristic (ROC) curve analysis.

**Results:**

FM patients had significantly higher levels of anti-β-LGB, ZO-1, LPS, and sCD14 than healthy controls (all *P* < 0.0001). In ME/CFS patients, levels of anti-β-LGB, ZO-1, LPS, and sCD14 were significantly higher than controls, but lower than in FM (all *P* < 0.01), while there was no significant difference in IL-1β level. In the FM and ME/CFS cohorts, both anti-β-LGB and ZO-1 correlated significantly with LPS and sCD14 (*P* < 0.001 for both). In the FM group, both anti-β-LGB and ZO-1 were correlated significantly with physical and mental health components on the SF-36 scale (*P* < 0.05); whereas IL-1β negatively correlated with the COMPASS-31 score (*P* < 0.05). In the ME/CFS cohort, ZO-1 was positively correlated with the COMPASS-31 score (*P* < 0.05). The ROC curve analysis indicated a strong ability of anti-β-LGB, ZO-1, LPS and sCD14 to predictively distinguish between FM and ME/CFS from healthy controls (P < 0.0001).

**Conclusion:**

Biomarkers of intestinal barrier function and inflammation were associated with autonomic dysfunction assessed by COMPASS-31 scores in FM and ME/CFS respectively. Anti-β-LGB antibodies, ZO-1, LPS, and sCD14 may be putative predictors of intestinal barrier dysfunction in these cohorts. Further studies are needed to assess whether these findings are causal and can therefore be applied in clinical practice.

## Introduction

Fibromyalgia (FM) and myalgic encephalomyelitis/chronic fatigue syndrome (ME/CFS) constitute a major public health issue worldwide, imposing a serious burden on patients, caregivers, and society, and exerting a substantial economic impact (1–4). Both are complex disabling multisystem disorders without an established aetiology characterized by a wide range of overlapping symptoms such as widespread pain, post-exertional fatigue, cognitive dysfunction, dysautonomia, and gastrointestinal complaints (5, 6). No simple diagnostic tests are available, nor any curative treatment (7, 8).

While FM and ME/CFS share common symptoms and biological abnormalities of unknown cause, a growing body of evidence suggests the existence of multiple pathophysiology mechanisms underlying the association between impaired gut barrier function and local and systemic inflammation in these conditions. Assessing gut barrier function in these conditions is challenging and has been a matter of debate for many years (9–11). Recently, it was proposed that increased intestinal permeability and gut dysbiosis allows the entry of bacterial endotoxins (reflected by high levels of specific anti-LPS antibodies) into the bloodstream and may trigger systemic inflammation and sustained immune hyperactivation (in the form of imbalances of inflammatory factors such as IL-1β, IL-6, TNF-α, IFN-γ, IL-10, IL-13, IL-16, IL-17A and C-reactive protein), contributing to the development and perpetuation of chronic widespread pain and post-exertional malaise in these illnesses (12–14).

Previous studies conducted in FM and ME/CFS have speculated about a possible association between intestinal function biomarkers (zonulin, LPS and sCD14) and compromised intestinal barrier integrity; however, more research is needed to understand the exact role and the connections between them (10, 15). Consequently, the growing evidence of a potential role of gut-brain axis in triggering neuroinflammation in FM and ME/CFS has identified several intestinal barrier function biomarkers which may contribute to the onset and illness severity. For instance, these studies have linked significant high levels of zonulin, LPS and its receptor sCD14 with increased intestinal permeability and microbial translocation in FM and ME/CFS (10, 15–22). Although the ability of these biomarkers to suggest the presence of compromised intestinal barrier in these conditions is well established, their use in clinical practice and research remains limited.

Although the potential involvement of an antibody-mediated autoimmune signature and illness severity has been proposed in the pathophysiology of these conditions (23), to date the circulating anti-beta-lactoglobulin antibody profile (anti-β-LGB) in FM and ME/CFS has not explored. Increased antibody production against LGB, a major allergen in whey (cow’s milk protein) has been observed in individuals with allergy and/or food intolerances and gastrointestinal complaints, which have also been reported in FM and ME/CFS (24, 25).

In addition, a growing number of studies linked the increased immune activation with the alteration of the gut microbiome composition in FM and ME/CFS (15, 23, 26–29), thereby suggesting that perturbed microbiome homeostasis may induce an imbalance in tolerance induction. An intestinal barrier dysfunction characterized by increased gut permeability and microbial translocation may lead to irritable bowel syndrome (IBS), which could impair tolerance the development of tolerance and instead contribute to exacerbation of symptoms in these conditions (11, 30, 31). Furthermore, the damaged intestinal barrier-induced immunity may contribute to neuroimmune dysfunction followed by the gradual activation of innate responses in the brain via the vagus nerve (neuroglial activation) and a reduction of energy-consuming activities in FM and ME/CFS (8, 32, 33).

Further investigations on the gut microbial composition and metabolomics profiling have shown noticeable reductions in the relative microbial abundance/diversity and the production of microbiota-derived metabolites in ME/CFS (i.e., probiotic *Bifidobacterium* species and butyrate-producing *Faecalibacterium*), possibly leading to the perturbed gut barrier function and increased bacteria translocation implicated in low-grade systemic inflammation (10, 14, 27, 34, 35). Similar alterations in gut microbiota composition have been reported in FM patients, who presented significant reductions in the relative abundance of certain short-chain fatty acid-producing bacteria, while higher relative abundance was reported for other organic acids (36–39).

Therefore, this study aimed (1) to explore whether patients with FM and ME/CFS have altered intestinal barrier function and inflammation by measuring circulating gut biomarkers and (2) to examine the relationships among these intestinal function biomarkers and self-reported outcome measures provided by the study participants.

## Methods

### Study design and participants

A proof-of-concept multicenter, cross-sectional, prospective case-control cohort study was conducted in 22 FM patients who met the 2010 ACR diagnostic criteria for FM (40) and 30 ME/CFS who fulfilled the 2011 ICC definition for ME/CFS (41). Subjects were recruited by clinicians from two outpatient referral centres (Hospital VIAMED Santa Angela de la Cruz, Seville, Spain and Vall d’Hebron Hospital, Barcelona, Spain) from February 2018 to March 2019. The sample comprised 26 age- and sex-matched sedentary healthy volunteers, who were recruited from each local community by word of mouth. Ten FM patients, 12 with ME/CFS and 13 sedentary healthy volunteers were recruited from the hospital in Sevilla and the rest from the local hospital in Barcelona so as to avoid sampling bias in the selection of the target population. The participants were Caucasian, from the same geographical area, and had a sedentary lifestyle with a similar Mediterranean dietary pattern at the time of inclusion.

The exclusion criteria for participation in the study included current or past diagnosis of autoimmune conditions (such as coeliac disease), food allergies and/or intolerances, haematological conditions, cardiovascular diseases, metabolic and endocrine disturbances (thyroid-related conditions), infectious diseases, neuropsychiatric disorders (psychosis/major depression). Individuals were also excluded if they had morbid obesity, were smokers, were pregnant and/or breast-feeding, or had history of substance misuse or any underlying symptoms that might influence the clinicians’ ability to distinguish between FM and ME/CFS diagnoses.

All study participants were informed of the research procedures and signed a written informed consent form prior to enrolment, in accordance with the 2013 Declaration of Helsinki. This study was approved by the local Research Ethics Committees (reference number: GutME-0634; on January 17, 2018). After providing consent, all participants underwent a clinical examination. Finally, data were analysed in an irreversibly anonymized fashion. A detailed summary of the participants’ demographic and clinical characteristics is shown in **Table 1**.

**Table 1:** Demographic and clinical characteristics of the study population.

Variable	FM (n = 22)	ME/CFS (n = 30)	HC (n = 26)	*P-*value ^a,b^
Age, years	57 ± 16	53 ± 10	51 ± 8	0.957/0.982
Gender, female (%)	16 (73)	24 (80)	20 (77)	0.954/0.971
BMI, kg/m^2^	23 ± 3.5	24 ± 5.1	24 ± 3.7	0.875/1.00
SBP, mmHg	120 ± 11	109 ± 14	118 ± 8	0.951/0.919
DBP, mmHg	80 ± 6	75 ± 10	68 ± 6	0.754/0.874
HR, bpm	70 ± 7	78 ± 9	72 ± 11	0.925/0.832
Marital status, n (%)				
Single	6 (27)	7 (23)	6 (23)	n.s.
Separated/divorced	4 (18)	5 (17)	5 (19)	n.s.
Married	12 (55)	18 (60)	15 (58)	n.s.
Illness duration, months				
48	2	2	n/a	n.s.
72	8	3	n/a	n.s.
≥ 120	12	25	n/a	n.s.
Family members affected, n (%)				
Yes	12 (55)	9 (30)	9 (34)	n.s.
No	10 (45)	21 (70)	17 (66)	n.s.
Medication use, n (%)				
Antidepressants				
Tricyclics				
Yes	4 (14)	6 (20)	0 (0)	n.s.
No	18 (86)	24 (80)	26 (100)	n.s.
Dual				
Yes	5 (23)	14 (47)	0 (0)	n.s.
No	17 (77)	16 (53)	26 (100)	n.s.
SSRI				
Yes	2 (10)	6 (20)	0 (0)	n.s.
No	20 (90)	24 (80)	26 (100)	n.s.
Anticonvulsants				
Yes	3 (14)	21 (70)	0 (0)	n.s.
No	19 (86)	9 (30)	26 (100)	n.s.
Tramadol				
Yes	9 (41)	10 (33)	0 (0)	n.s.
No	13 (59)	20 (67)	26 (100)	n.s.
Major opioids				
Yes	2 (10)	3 (10)	0 (0)	n.s.
No	20 (90)	27 (90)	26 (100)	n.s.
Anxiolytics/sedatives				
Yes	1 (4)	6 (20)	0 (0)	n.s.
No	21 (96)	24 (80)	26 (100)	n.s.
NSAIDs				
Yes	7 (32)	14 (47)	0 (0)	n.s.
No	15 (68)	16 (53)	26 (100)	n.s.
Other analgesics				
Yes	10 (45)	19 (63)	0 (0)	n.s.
No	12 (55)	11 (37)	26 (100)	n.s.
Measures				
FIS-40				
Global score (0-160)	113 ± 19.6	140.7 ± 19.4	15.6 ± 5.4	< 0.0001
Physical	32 ± 6	36.7 ± 3.4	4.4 ± 0.3	< 0.0001
Cognitive	30 ± 5	37 ± 5.9	4.1 ± 0.7	< 0.0001
Psychosocial	51 ± 9	67 ± 12	7.1 ± 1.9	< 0.0001
COMPASS-31				
Global score (0-100)	64.5 ± 2.8	65.3 ± 1.4	6.8 ± 1.1	< 0.0001
Orthostatic intolerance	31 ± 2.8	30.6 ± 6.4	4 ± 1.1	< 0.0001
Vasomotor	2.4 ± 1.2	2.5 ± 0.4	0.0 ± 0.0	< 0.0001
Secretomotor	10.5 ± 1.7	10.7 ± 2.3	1.3 ± 0.4	< 0.0001
Gastrointestinal	12.9 ± 3.1	13.4 ± 3.8	0.7 ± 0.2	< 0.0001
Bladder	4.2 ± 3.2	4.4 ± 3.2	0.0 ± 0.0	< 0.0001
Pupillomotor	3.5 ± 0.9	3.7 ± 0.9	0.8 ± 0.2	< 0.0001
PSQI				
Global score (0-21)	5.0 ± 2.0	18.0 ± 3.1	4.0 ± 2.0	< 0.0001
Subjective sleep quality	1.0 ± 0.6	3.0 ± 0.6	1.0 ± 0.6	< 0.0001
Sleep latency	1.0 ± 0.6	3.0 ± 0.8	1.0 ± 0.8	< 0.0001
Sleep duration	0.5 ± 0.4	2.0 ± 0.4	1.0 ± 0.4	< 0.0001
Habitual sleep efficiency	0.5 ± 0.4	3.0 ± 0.3	0.0 ± 0.0	< 0.0001
Sleep disturbances	1.0 ± 0.5	2. 0 ± 0.3	1.0 ± 0.3	< 0.0001
Sleeping medication	1.0 ± 0.5	3.0 ± 0.3	0.0 ± 0.0	< 0.0001
Daytime dysfunction	0.0 ± 0.0	3.0 ± 0.5	0.0 ± 0.0	< 0.0001
SF-36				
Physical functioning	14 ± 0.8	18.3 ± 5.5	100 ± 0.0	< 0.0001
Physical role functioning	0.0 ± 0.0	0.0 ± 0.0	98 ± 7.9	< 0.0001
Bodily pain	9 ± 0.8	11 ± 9.6	100 ± 0.0	< 0.0001
General health perception	12 ± 1.9	17 ± 11.8	80 ± 15.7	< 0.0001
Vitality	10 ± 0.9	5 ± 0.9	90 ± 12.4	< 0.0001
Social role functioning	14 ± 0.7	20 ± 3.9	100 ± 0.0	< 0.0001
Emotional role functioning	25 ± 3.7	26 ± 4.8	100 ± 0.0	< 0.0001
Mental health	17 ± 0.6	44 ± 12.3	92 ± 8.1	< 0.0001
PCS	24.2 ± 0.1	20.9 ± 1.0	57.0 ± 0.4	< 0.0001
MCS	7.6 ± 0.2	18.3 ± 2.5	54.3 ± 0.6	< 0.0001

Data are given as means ± standard error of the mean (SEM) for continuous variables, and as number of cases (percentages) for categorical variables, as appropriate (unless otherwise specified). P-values from Mann-Whitney U-test for continuous variables and from Fisher’s exact test for categorical variables (gender, marital status, family background, medications). Bold values denote statistical significance at P < 0.05 between each cohort (^a^FM and ^b^ME/CFS) with healthy controls. BMI, body mass index; SBP, systolic blood pressure; DBP, diastolic blood pressure; HR, heart rate; SSRI, selective serotonin reuptake inhibitors; NSAIDs, non-steroidal anti-inflammatory drugs; FIS-40, 40-item fatigue impact scale; COMPASS-31, composite autonomic symptom score; PSQI, Pittsburgh sleep quality index; SF-36, 36-item short-form health survey; PCS, physical health component summary scores; MCS, mental health component summary scores; n.s., not significant.

### Data collection and clinical outcomes measures

Participants were asked to complete validated self-reported questionnaires under the supervision of two trained investigators, who ensured compliance. These questionnaires included the Fatigue Impact Scale (FIS-40), the Composite Autonomic Symptom Score (COMPASS-31), the Pittsburgh Sleep Quality Index (PSQI), and the Short Form 36-item (SF-36) health survey, which were used to compile data on participants’ demographic characteristics and current health status.

### Fatigue impact scale

Fatigue was assessed by the Fatigue Impact Scale (FIS-40), a self-administered 40-item questionnaire which includes three subscales (scored from 0 to 4) reflecting the perceived feeling of fatigue in physical (10 items), cognitive (10 items), and psychosocial functions (20 items). The sum of the three scales yields a global score ranging from 0 to 160. Higher scores indicate more functional limitations resulting from fatigue; scores above 120 points are taken to indicate severe fatigue, while scores of 120 points or less are taken to reflect mild/moderate fatigue (42).

### Composite autonomic symptom score

The frequency and severity of autonomic symptoms were evaluated by using the validated self-administered 31-item Composite Autonomic Symptom Score (COMPASS-31), comprising six main domains: orthostatic intolerance (4 items), vasomotor (3 items), secretomotor (4 items), gastrointestinal (12 items), bladder (3 items), and pupillomotor systems (5 items). The overall COMPASS-31 score ranges from 0 to 100, with higher scores indicating worse autonomic symptoms (43).

### Pittsburgh sleep quality index

Sleep disturbances were assessed by the standardized, self-administered 19-item Pittsburgh Sleep Quality Index (PSQI) questionnaire, which comprises seven components of sleep quality assessed over a one-month interval (scored from 0 to 3): subjective sleep quality, latency, sleep duration, habitual sleep efficiency, sleep perturbations, use of sleeping medication, and daytime dysfunction. The global PSQI score can range from 0 to 21 points, with score above 5 representing poorer subjective sleep quality (44).

### Short form 36-item health survey

Participants’ general physical and mental health was assessed using the short form 36-item (SF-36) questionnaire, a 36-item self-report health survey conducted over a 4-week period. The SF-36 comprises eight health domains, focusing on limitations in physical activities due to health issues, limitations in social activities due to physical or/and emotional problems, limitations in everyday activities due to health and/or psychological problems, bodily pain, general mental health (including psychological distress and well-being), vitality and overall health perceptions. The eight domains were weighted and summarized in physical component summary (PCS) scores and mental component summary (MCS) scores ranging from 0 to 100. Higher scores indicate better health-related physical and mental quality of life (45).

### Collection of blood samples and processing

Fasting blood samples were collected from each participant directly in K_2_EDTA tubes (Vacutainer, BD Biosciences, Madrid, Spain) by venipuncture upon confirmation of the diagnosis. Plasma samples were obtained by centrifugation at 2,000 x *g* for 15 minutes at 4°C within 1 hour of the blood collection; then they were collected immediately and frozen in aliquots at –80°C until further analysis.

### Quantification of intestinal barrier function biomarkers

Plasma ZO-1 (Catalog #: MBS706368, MyBioSource, San Diego, CA), LPS (Catalog #: MBS266722, MyBioSource, San Diego, CA), sCD14 (Catalog #: RK01060, Abyntek Biopharma, Vizcaya, Spain), and IL-1β (Catalog #: MBS2510385, MyBioSource, San Diego, CA) were measured using commercially available ELISA kits according to the manufacturers’ instructions. Circulating human anti-β-lactoglobulin antibody levels (IgG isotypes) were assayed using a validated home-made ELISA protocol as detailed below. All the plasma samples were measured in blind duplicates for each biomarker. For all protocols, absorbance at 450 nm with 570 nm correction was measured in a microplate reader, and corrected absorbance was interpolated in each standard curve to determine the concentrations (Sigma Plot).

### Detection of human anti-β-lactoglobulin antibody levels

Circulating anti-β-lactoglobulin antibody levels (IgG isotypes) were tested using a standard direct ELISA protocol (46). Briefly, microtitre 96-well polystyrene plates (Nunc, Roskilde, Denmark) were coated with 1 μg/μL β-lactoglobulin from cow’s milk as target protein (Cat # L7880; Sigma Aldrich, Madrid, Spain) diluted in 50 mM Na_2_CO_3_/NaHCO_3_ buffer (pH 9.6) at 4°C overnight. After blocking with 1X phosphate-buffered saline containing 0.05% Tween-20 (PBS-T) and 3% BSA for 90 minutes at room temperature, the pre-coated plates were incubated with the human plasma samples diluted in blocking buffer (dilution from 1:200 to 1:2000) for two hours at room temperature. Plates were washed out three times with PBS-T for five minutes and then each well was incubated with an HRP-conjugated mouse anti-human IgG1 Fc secondary antibody at dilution 1:5000 (Cat # A-10648; Thermo Fisher Scientific, MA, USA) for three hours at room temperature. Between incubations, plates were washed three times with PBS-T. Plates were revealed using TMB substrate (Thermo Fisher Scientific, MA, USA) for 30 minutes at room temperature in the dark and then 50 μl of stop solution (2M sulphuric acid) was added to each well. The absorbance (O.D.) was read immediately at 450 nm with 570 nm correction using a microplate reader (Varioskan Flash, Thermo Electron Corporation, NH, USA). The results were given as antibody levels per ng/ml on a standard curve with anti-human beta-LGB chimera monoclonal antibody (clone 25I2; CABT-L2429; Creative Diagnostics, NY, USA) considering the dilution of each sample. The standard curve was prepared using serial dilutions from 8000 ng/ml to 31.25 ng/ml and included five standard concentrations within the indicated range. Based on the signal-to-noise ratio of assay, the limit of detection was 1.96 ng/ml. In each ELISA assay, endogenous negative and positive controls of human plasma samples were included. Negative controls were tested from healthy donors who had not ingested dairy products for at least one year. Positive controls were assayed from patients recently diagnosed with celiac disease, before the start of treatment, and who habitually ingested dairy products.

### Statistical analysis

All the data obtained were checked for normality with the Kolmogorov-Smirnov and Shapiro-Wilk tests. Normally distributed data were presented as means ± standard error of the mean (SEM). Statistical analysis of non-parametric data in the three study groups was performed using an analysis of non-parametric data evaluated by the Kruskal-Wallis test and compared by Dunn’s multiple comparison test. Scale and subscale scores were examined using the non-parametric Mann-Whitney *U* test (two-group comparisons). Fisher’s exact test was used to compare the frequency of the reported categorical variables between the groups. Box plots and ROC curves were generated using GraphPad Prism software. The area under the curve (AUC) was calculated so as to compare the overall diagnostic accuracy of gut biomarkers for predicting FM and ME/CFS. Cut-off values with the highest accuracy were selected as the diagnostic cut-off points. Correlations were analysed using Spearman’s test with the R package. Statistical significance was set at *P* < 0.05 (two-tailed). Only adjusted *P*-values presenting significant differences are shown. Statistical analyses were performed using R software version 4.0.2 (R Foundation for Statistical Computing, Vienna, Austria) and GraphPad Prism version 9.5.1 for Windows (GraphPad software, Boston, MA, USA).

## Results

### Baseline demographics and clinical characteristics of the study participants

Seventy-eight participants, comprising 22 FM patients, 30 ME/CFS patients and 26 healthy-matched controls were included in the study. **Table 1** shows the demographics and clinical characteristics of the study population. No significant differences were observed for age, gender, BMI, hemodynamic variables, marital status, illness duration, family background, and concomitant medication between the study cohorts from each site. In this group, most patients with FM (n = 12) and ME/CFS (n = 25) had an illness duration of more than 120 months (10 years), which was self-reported as long-lasting fatigue and chronic pain (*data not shown*). More than half of the patients reported frequent medication use, including analgesics, non-steroidal anti-inflammatories, anticonvulsants, anxiolytics and antidepressants, but none of the controls were taking medication.

The clinical assessment based on self-reported outcome measures showed that patients with FM and ME/CFS had significantly higher scores on the FIS-40, COMPASS-31 and PSQI questionnaires than controls (all *P* < 0.0001), whereas healthy controls had significantly higher SF-36 scores than the patients’ groups (all *P* < 0.0001).

### Profile of biomarkers of intestinal barrier function and inflammation

**Figure 1:**
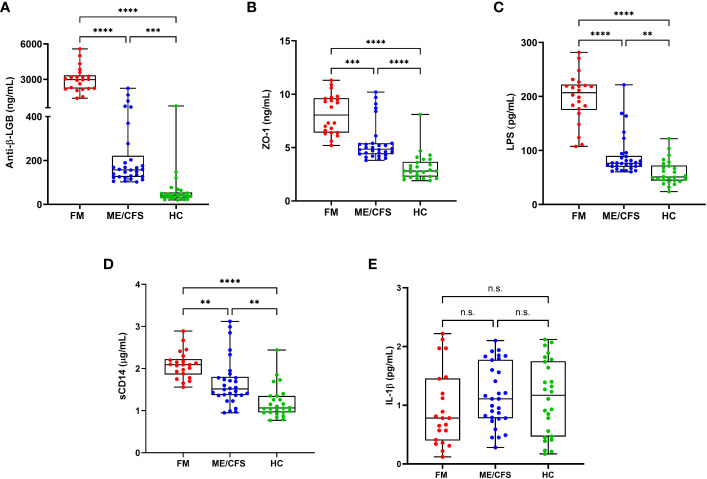
Circulating biomarkers of intestinal permeability, bacterial translocation and inflammation in the study participants. Plasma levels of anti-β-LGB **(A)**, ZO-1 **(B)**, LPS **(C)**, sCD14 **(D)**, and IL-1β **(E)** in patients with FM (n = 22), ME/CFS (n = 30) and healthy controls (n = 26). Each dot denotes a single participant. Values are shown as mean ± SEM of duplicates and are representative of two independent experiments. The box extends from the 25^th^ to 75^th^ percentiles, the line represents the mean, and the whiskers indicate the range of minimum and maximum values. Significance at **P < 0.01, ***P < 0.001, and ****P < 0.0001 was calculated using the Kruskal-Wallis signed-rank test on normalized data. Anti-β-LGB, anti-beta-lactoglobulin antibodies; ZO-1, zonulin-1; LPS, lipopolysaccharides; sCD14, soluble CD14; IL-1β, interleukin-1 beta. n.s., not significant

The measurement of intestinal biomarkers proposed in this study revealed that FM patients had higher presence of increased gut permeability and microbial translocation than ME/CFS patients and matched controls. As shown in **Figure 1**, plasma levels of intestinal barrier function biomarkers and inflammation were as follows: IgG anti-β-LGB antibodies (A), ZO-1 (B), LPS (C), sCD14 (D), and IL-1β (E) in individuals with FM, ME/CFS, and healthy controls. FM patients had significantly higher levels of IgG anti-β-LGB antibodies, ZO-1, LPS and sCD14 than ME/CFS and controls (*P* < 0.001 for all). In ME/CFS patients, plasma levels of anti-β-LGB, ZO-1, LPS, and sCD14 were significantly higher than in controls (*P* < 0.01), but lower than in FM cases (*P* < 0.001). There were no significant differences in IL-1β levels in patients with FM and ME/CFS and healthy controls.

### Correlation analysis between intestinal function biomarkers and self-reported outcome measures

**Figure 2:**
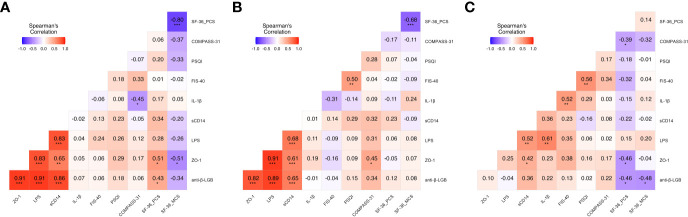
Heatmap depicting color-coded Spearman’s correlation coefficients of circulating intestinal barrier function biomarkers and clinical outcome measures in the study participants. Correlation analysis of patients with FM **(A)**, ME/CFS **(B)** and healthy controls **(C)** were evaluated using Spearman’s rank correlation test and FDR-adjusted *P* < 0.05. Pairwise Spearman’s rank correlation coefficients (rho) are depicted for each correlation and is presented by color intensity scale (at the top left of each panel). Heat color show standardized Z-scores (adjusted rho) across biomarkers and outcome measures. The color intensity is proportional to the strength of the association (rho value) ranging from red (positive correlations) to blue (negative correlations). Statistical significance was assessed using the Kruskal-Wallis test. FDR was calculated using Benjamini-Hochberg method. Statistical significance was set at *P < 0.05, **P < 0.01, and ***P < 0.001. Anti-β-LGB, anti-beta-lactoglobulin antibodies; ZO-1, zonulin-1; LPS, lipopolysaccharides; sCD14, soluble CD14; IL-1β, interleukin-1-beta.

Correlations between the proposed biomarkers of intestinal barrier function and inflammation and self-reported outcome measure scores in the study cohorts are displayed in **Figure 2**. Briefly, in the FM cohort, ZO-1 was significantly correlated with anti-β-LGB antibodies (r = 0.91; *P* < 0.001), LPS (r = 0.83; *P* < 0.001) and sCD14 (r = 0.65; *P* < 0.01), and with physical and mental health component scores on the SF-36 questionnaire (r = 0.51 and r = -0.51; both *P* < 0.05) respectively. In this cohort, anti-β-LGB was strongly correlated with ZO-1 (r = 0.91; *P* < 0.001), LPS (r = 0.91; *P* < 0.001), and sCD14 (r = 0.86; *P* < 0.001) and also with physical health component scores on the SF-36 questionnaire (r = 0.43; *P* < 0.05). In contrast, IL-1β negatively correlated with overall COMPASS-31 scores (r = -0.45; *P* < 0.05) (**Figure 2A**).

Analysis of the ME/CFS cohort showed significant positive correlations between ZO-1 and anti-β-LGB (r = 0.82; *P* < 0.001), LPS (r = 0.91; *P* < 0.001) and sCD14 (r = 0.61; *P* < 0.001), and COMPASS-31 scores (r = 0.45; *P* < 0.05), whereas anti-β-LGB was positively correlated with ZO-1 (r = 0.82; *P* < 0.001), LPS (r = 0.89; *P* < 0.001) and sCD14 (r = 0.65; *P* < 0.001) (**Figure 2B**).

In healthy controls, ZO-1 was positively and significantly correlated with sCD14 (r = 0.42; *P* < 0.05) and opposed with physical healthy component scores on the SF-36 questionnaire (r = -0.46; *P* < 0.05); while anti-β-LGB was negatively correlated with physical and mental health component scores on the SF-36 questionnaires (r = -0.46 and r = -0.48; both *P* < 0.05), respectively (**Figure 2C**). Multipanel scatter dot plots for statistically significant correlations between intestinal barrier function biomarkers and self-reported outcome measures in FM, ME/CFS and healthy controls are depicted (**Supplementary Figs S1-S3**).

### ROC analysis for each intestinal barrier function biomarker in FM and ME/CFS

**Figure 3:**
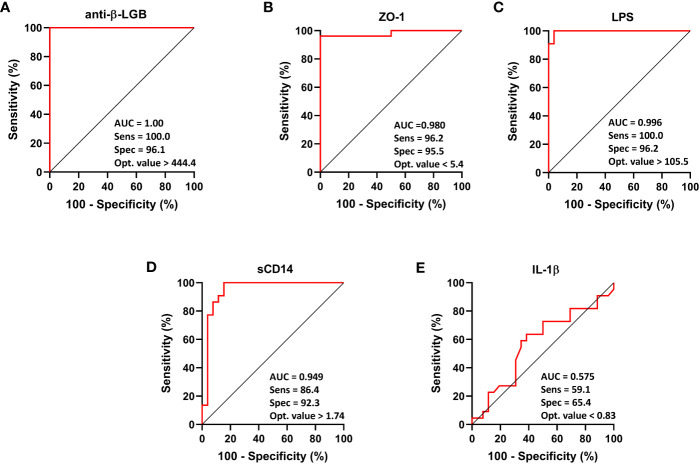
ROC curve analysis of each intestinal barrier function biomarker to discriminate FM patients from healthy controls. IgG anti-β-LGB antibodies **(A)**, ZO-1 **(B)**, LPS **(C)**, sCD14 **(D)**, and IL-1β **(E)**. ROC curves were used to explore the accuracy of each biomarker to discriminate between FM subjects and healthy controls. Cut-off values are shown for each biomarker with their respective sensitivity, specificity and optimal value. AUC values close to 1 indicate that a high true positive rate was achieved with false positive rate (ideal performance), while AUC values close to 0.5 indicate random performance. Anti-β-LGB, anti-beta-lactoglobulin antibodies; ZO-1, zonulin-1; LPS, lipopolysaccharides; sCD14, soluble CD14; IL-1β, interleukin-1-beta.

**Figure 4:**
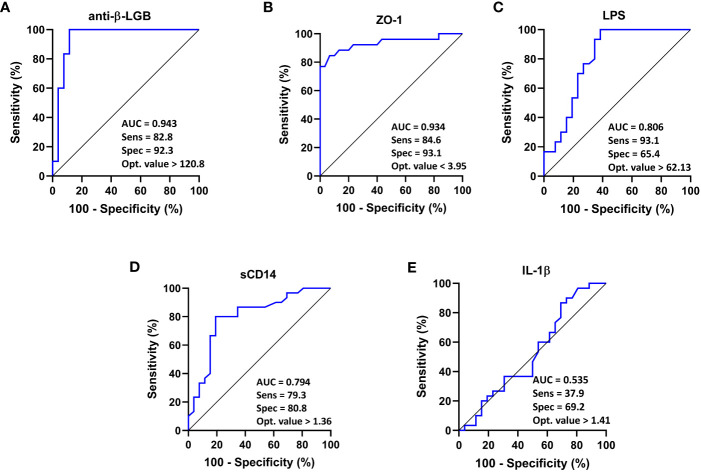
ROC curve analysis of each intestinal barrier function biomarker to discriminate ME/CFS patients from healthy controls. IgG anti-β-LGB antibodies **(A)**, ZO-1 **(B)**, LPS **(C)**, sCD14 **(D)**, and IL-1β **(E)** are displayed. ROC curves were used to analyze the accuracy of each biomarker to discriminate between ME/CFS subjects and healthy controls. Cut-off values are shown for each biomarker with their respective sensitivity, specificity and optimal value. AUC values close to 1 indicate that a high true positive rate was achieved with false positive rate (ideal performance), while AUC values close to 0.5 indicate random performance. Anti-β-LGB, anti-beta-lactoglobulin antibodies; ZO-1, zonulin-1; LPS, lipopolysaccharides; sCD14, soluble CD14; IL-1β, interleukin-1-beta.

Analyses of the diagnostic power of each circulating gut function biomarker with regard to predictively distinguishing FM and ME/CFS from healthy controls are displayed in **Figures 3** and **4** respectively. As shown in the ROC curve analysis for FM, compared to the reference, anti-β-LGB (AUC = 1.00; 95% CI: 1.00-1.00; *P* < 0.0001), ZO-1 (AUC = 0.980; 95% CI: 0.94-1.00; *P* < 0.0001), LPS (AUC = 0.996; 95% CI: 0.98-1.00; *P* < 0.0001), and sCD14 (AUC = 0.949; 95% CI: 0.88-1.00; *P* < 0.0001) were able to distinguish between FM patients and healthy controls as demonstrated by the AUC values using a univariate model (**Figures 3A-E**).

ROC curve analysis for ME/CFS showed that compared to the reference, anti-β-LGB (AUC = 0.943; 95% CI: 0.86-1.00; *P* < 0.0001), ZO-1 (AUC = 0.934; 95% CI: 0.86-1.00; *P* < 0.0001), LPS (AUC = 0.806; 95% CI: 0.68-0.93; *P* < 0.0001), and sCD14 (AUC = 0.794; 95% CI: 0.68-0.92; *P* < 0.0001) were able to distinguish between ME/CFS patients and healthy controls as demonstrated by the AUC values using a univariate model (**Figures 4A-E**).

**Figure 5:**
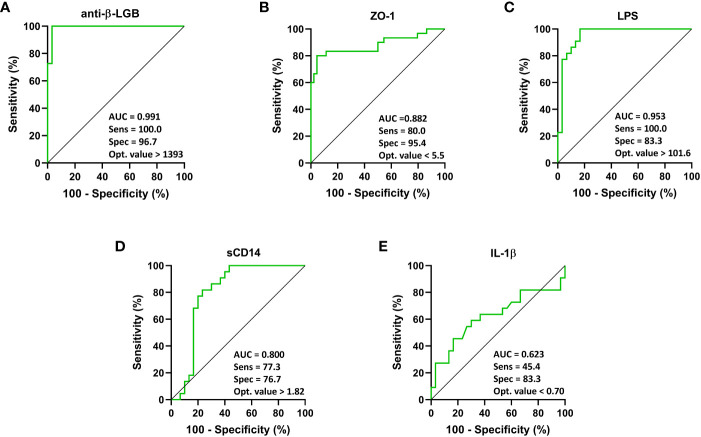
ROC curve analysis of each intestinal barrier function biomarker to discriminate individuals with FM from ME/CFS. IgG anti-β-LGB antibodies **(A)**, ZO-1 **(B)**, LPS **(C)**, sCD14 **(D)**, and IL-1β **(E)** are displayed. ROC curves were used to analyze the accuracy of each biomarker to discriminate between ME/CFS subjects and healthy controls. Cut-off values are shown for each biomarker with their respective sensitivity, specificity and optimal value. AUC values close to 1 indicate that a high true positive rate was achieved with false positive rate (ideal performance), while AUC values close to 0.5 indicate random performance. Anti-β-LGB, anti-beta-lactoglobulin antibodies; ZO-1, zonulin-1; LPS, lipopolysaccharides; sCD14, soluble CD14; IL-1β, interleukin-1-beta.

Also, an ROC curve analysis to predictively distinguish patients with FM and ME/CFS is shown in **Figure 5**. It showed that compared to the reference, anti-beta-LGB (AUC = 0.991; 95% CI: 0.97 to 1.00; *P* < 0.0001), ZO-1 (AUC = 0.882; 95% CI: 0.78 to 0.97; *P* < 0.0001), LPS (AUC = 0.953; 95% CI: 0.89 to 1.00; *P* < 0.0001), and sCD14 (AUC = 0.800; 95% CI: 0.67 to 0.92; *P* = 0.0002) were able to distinguish between patients with FM and ME/CFS as demonstrated by the AUC values using a univariate model (**Figures 5A-E**).

## Discussion

This is a proof-of-concept study to investigate the relationship between circulating intestinal function biomarkers and inflammation and self-reported clinical symptoms in Spanish patients with FM and ME/CFS, and also to evaluate the suitability of these gut barrier function biomarkers as potential suggestive predictors of diagnosis in these cohorts which replicate prior studies (14–16, 19, 47–51). Our findings corroborate those of previous studies (9, 12, 14, 19, 47, 48, 52) reporting the presence of significantly increased levels of suggestive biomarkers of intestinal permeability (IgG anti-β-LGB antibodies and ZO-1), and microbial translocation (LPS and sCD14) in FM and ME/CFS compared to healthy controls. Interestingly, these biomarkers were markedly higher in individuals with FM than in those with ME/CFS.

Further analysis indicated that the proposed novel intestinal permeability biomarkers (anti-β-LGB antibodies and ZO-1) significantly correlated with indices of microbial translocation (LPS and sCD14) in FM and ME/CFS. In addition, these measures were correlated with scores of self-reported outcome measures determined by COMPASS-31 and SF-36 questionnaires in FM and ME/CFS. Specifically, in FM the IL-1β levels were associated with measures of physical and mental health components on the SF-36 questionnaire; whereas the frequency and severity of autonomic symptoms evaluated by COMPASS-31 scores was positively correlated with the ZO-1 in the ME/CFS cohort. Further analysis of covariates indicated a significant correlation between age and anti-β-LGB and ZO-1, as well as LPS and sCD14 in ME/CFS patients. Finally, the ROC curve analysis of the diagnostic accuracy of the biomarkers measured demonstrated a high predictive capacity of anti-β-LGB, ZO-1, LPS and sCD14 for distinguishing FM and ME/CFS cases from healthy controls.

Recently, a growing number of studies have reported gut dysbiosis and increased intestinal permeability in ME/CFS (9, 10, 53), while in FM this phenomenon is still to be confirmed. The study by Goebel et al. of FM patients with complex regional pain syndromes who reported alterations of gut barrier integrity in the form of increased gastroduodenal and small intestinal permeability found that these conditions coincided with typical IBS symptoms which were recorded in up to 18% of FM patients (16, 54). Although few overlapping mechanisms explaining the high prevalence of GI symptoms in FM patients have been proposed, immune activation and neurotransmitter disruption have recently attracted attention (54).

Additionally, in ME/CFS it has been shown that elevated levels of bacterial wall components such as LPS, followed by an increased presence in the intestine of Gram-negative bacteria and also plasmacytoid dendritic cells as uniquely immunoreactive to antibodies against HERV proteins that damage the gut epithelial barrier and infiltrate in the bloodstream can provoke an immune response, ultimately leading to the establishment of low-grade chronic systemic inflammation (10, 55). Separately, the significant increases in Gram-positive facultative anaerobic bacteria reported in ME/CFS, including D-lactic acid-producing Enterococcus and Streptococcus spp., suggest that these bacteria are a more significant source of lactate than Gram-negative Escherichia coli. Gram-positive bacteria may thus contribute to the cognitive symptoms and also the mitochondrial dysfunction resulting from the lactic acidosis in this cohort (53).

Although intestinal damage may arise due to various pathomechanisms and may involve several factors, the evidence to date highlights its involvement in the context of FM and ME/CFS. For instance, disturbances in the gut wall may increase intestinal permeability, which can induce inflammatory changes that lead to comorbid chronic diseases. Loss of gut barrier integrity may contribute to bacterial translocation into the systemic circulation, followed by increased levels of autoantibodies IgA and IgM against LPS and more severe ME/CFS symptoms (22). In addition, changes in the gut microbiome in ME/CFS, with noticeable decreased bacterial diversity (in particular, a reduction in the relative abundance of members belonging to the Firmicutes phylum) may increase the predisposition to gut inflammation (14).

In the present study, both FM and ME/CFS patients had significantly higher levels of intestinal function biomarkers that indicate increased gut permeability and bacterial translocation than healthy controls. These observations are consistent with those of an earlier study that reported the presence of gut dysbiosis as demonstrated by increased levels of LPS, sCD14, endotoxins and lipid binding proteins (LBPs), which was positively correlated with illness severity in patients with chronic fatigue (48). A recent study, however, despite showing the presence of the antibody-induced responses to both microbial and dietary antigens along with greater epithelial cell damage and turnover rate confirmed by higher FABP-2 levels, failed to report any significant differences in LBPs or sCD14 levels due to a suppressed anti-microbial response in ME/CFS compared with controls (50). Interestingly another study, which reported increased levels of LPS and sCD14 along with some other biomarkers not included in this analysis (such as LBP, I-FABP, MCP-1 and C-reactive protein) was able to correctly discriminate between ME/CFS and controls with a cross-validation accuracy of 82.9% (14).

The findings reported here are of particular interest, because they confirm an association between the changes in indicators of increased intestinal permeability and bacterial translocation and their association with clinical outcomes measures in FM and ME/CFS. Besides, the present data add to the evidence that a gut barrier integrity injury may be involved in the pathophysiology of these illnesses, even if it is not always detectable.

To our knowledge, this is the first confirmatory study to explore the use of intestinal barrier function biomarkers related to increased gut permeability, such as anti-β-LGB antibodies in a Spanish FM and ME/CFS cohort. The main strength of the study is that data on the participants were obtained from a well-phenotyped cohort of Spanish FM and ME/CFS patients from two Spain outpatient referral centres, applying updated diagnostic case criteria and validated self-reported symptom questionnaires in these conditions. However, this study has several limitations, including its small sample size, its cross-sectional nature, and limited measures of inflammatory cytokine/chemokine and growth factor markers which are unable to establish causation between disrupted gut mucosal barrier and inflammation severity status.

In addition, self-reporting outcome measures do not use “*in vivo*” differential urinary multi-sugar excretion test for small bowel and colonic permeability assessment. It should be also noted that the bacterial DNA load assessed by culturing bacteria directly from blood and stool was not measured in these populations, and so the presence of potential infection cannot be conclusively ruled out, or its potential influence on the bacterial translocation biomarkers LPS and sCD14 (21). Finally, no information was available on other confounding factors related to lifestyle habits, previous infections, use of high-dose antibiotics, concomitant drugs, psychological stress, air pollutants, and others comorbid health conditions such as IBS, and/or anxiety/depression.

Further multisite longitudinal studies with larger numbers of participants who are representative of the general population should be conducted to confirm the observations reported here. Future studies should also expand the use of other validated inflammatory biomarkers in stool and blood in order to fully define the specific role of disturbed intestinal barrier function and inflammation in FM and ME/CFS. These studies should also include lactulose breath testing (SIBO) by collecting longitudinal faecal samples to explore gender-dependent microbiota composition in these conditions (29, 56).

In conclusion, by demonstrating the associations between the putative biomarkers of gut barrier dysfunction and bacterial translocation, and self-reported clinical outcomes assessed by the COMPASS-31 score, our findings add to the existing evidence of the potential role of increased intestinal permeability in FM and ME/CFS. Future research should aim to confirm the applicability of these findings in clinical practice by targeting gastrointestinal complaints in FM and ME/CFS and assessing the usefulness of interventions focused on the restoring gut microbiota homeostasis and enhancing intestinal barrier function. If future studies show this strategy to be valid, it may offer new therapeutic benefit and provide an opportunity to reduce gastrointestinal symptoms and restore the quality of life of these patients.

## Data availability statement

The original contributions presented in the study are included in the article/**Supplementary Material**. Further inquiries can be directed to the corresponding authors.

## Ethics statement

This study was approved by the local Research Ethics Committees (reference number: GutME-0634; on January 17, 2018). The studies were conducted in accordance with the local legislation and institutional requirements. The participants provided their written informed consent to participate in this study.

## Author contributions

Conception and design of the study: FM, MB-S, JA-M, OC, and JC-M. Biostatistical analysis: BL, JCD, JJ, and JC-M. Acquisition of data: FM, PZ, and JC-M. Analysis and interpretation of data: FM, BL, JCD, JJ, and JC-M. Drafting the manuscript: FM, AG-C, JJ and JC-M. Review and editing of manuscript: FM, MA, BL, JS, JJ, and JC-M. Supervision: MB-S, JJ and JA-M. Project administration: FM, PZ, and JC-M. All authors contributed to the article and approved the submitted version.

## Supplementary material

Supplementary Figure 1 Multipanel scatter dot plots depicting the statistically significant between intestinal barrier function biomarkers and self-reported outcome measures in fibromyalgia patients. Each dot corresponds to an individual. Spearman’s correlation scatter plots with linear regression (black line) and the 95% confidence interval (brown band) was used to calculate the association. Square Spearman’s rank correlation coefficient (rho^2^) and statistically significant p-values are shown in each panel.

Supplementary Figure 2 Multipanel scatter dot plots depicting the statistically significant between intestinal barrier function biomarkers and self-reported outcome measures in ME/CFS patients. Each dot corresponds to an individual. Spearman’s correlation scatter plots with linear regression (black line) and the 95% confidence interval (brown band) was used to calculate the association. Square Spearman’s rank correlation coefficient (rho2) and statistically significant p-values are shown in each panel.

Supplementary Figure 3 Multipanel scatter dot plots depicting the statistically significant between intestinal barrier function biomarkers and self-reported outcome measures in healthy controls. Each dot corresponds to an individual. Spearman’s correlation scatter plots with linear regression (black line) and the 95% confidence interval (brown band) was used to calculate the association. Square Spearman’s rank correlation coefficient (rho2) and statistically significant p-values are shown in each panel.

## References

[1] D'Onghia M Ciaffi J Ruscitti P Cipriani P Giacomelli R Ablin JN. The economic burden of fibromyalgia: A systematic literature review. Semin Arthritis Rheum (2022) 56:152060. doi: 10.1016/j.semarthrit.2022.152060 35849890

[2] Nacul L Authier FJ Scheibenbogen C Lorusso L Helland IB Martin JA. European network on myalgic encephalomyelitis/chronic fatigue syndrome (EUROMENE): Expert consensus on the diagnosis, service provision, and care of people with ME/CFS in Europe. Medicina (Kaunas) (2021) 57:510. doi: 10.3390/medicina57050510 34069603 PMC8161074

[3] Araja D Berkis U Lunga A Murovska M. Shadow burden of undiagnosed myalgic encephalomyelitis/chronic fatigue syndrome (ME/CFS) on society: Retrospective and prospective-in light of COVID-19. J Clin Med (2021) 10. doi: 10.3390/jcm10143017 PMC8303374 34300183

[4] Pheby DFH Araja D Berkis U Brenna E Cullinan J de Korwin JD. The role of prevention in reducing the economic impact of ME/CFS in Europe: A report from the socioeconomics working group of the European network on ME/CFS (EUROMENE). Medicina (Kaunas) (2021) 57. doi: 10.3390/medicina57040388 PMC8073750 33923830

[5] Natelson BH. Myalgic encephalomyelitis/chronic fatigue syndrome and fibromyalgia: Definitions, similarities, and differences. Clin Ther (2019) 41:612–18. doi: 10.1016/j.clinthera.2018.12.016 PMC6589349 30795933

[6] McKay PG Martin CR Walker H Fleming M. Chronic fatigue syndrome (CFS)/Myalgic Encephalomyelitis (ME) and Fibromyalgia (FM): the foundation of a relationship. Br J Pain (2021) 15:26–39. doi: 10.1177/2049463719875164 PMC7882776 33633851

[7] Giorgi V Sirotti S ROmano ME Marotto D Ablin JN Salaffi F. Fibromyalgia: one year in review 2022. Clin Exp Rheumatol (2022) 40:1065–72. doi: 10.55563/clinexprheumatol/if9gk2 35748720

[8] Tate WP Walker MOM Peppercorn K Blair ALH Edgar CD. Towards a better understanding of the complexities of myalgic encephalomyelitis/chronic fatigue syndrome and long COVID. Int J Mol Sci (2023) 24. doi: 10.3390/ijms24065124 PMC10048882 36982194

[9] Varesi A Deumer US Ananth S Ricevuti G. The emerging role of gut microbiota in myalgic encephalomyelitis/chronic fatigue syndrome (ME/CFS): Current evidence and potential therapeutic applications. J Clin Med (2021) 10. doi: 10.3390/jcm10215077 PMC8584653 34768601

[10] Konig RS Albrich WC Kahlert CR Bahr LS Lober U Vernazza P. The gut microbiome in myalgic encephalomyelitis (ME)/chronic fatigue syndrome (CFS). Front Immunol (2021) 12:628741. doi: 10.3389/fimmu.2021.628741 35046929 PMC8761622

[11] Garofalo C Cristiani CM Ilari S Passacatini LC Malafoglia V Viglietto G. Fibromyalgia and irritable bowel syndrome interaction: A possible role for gut microbiota and gut-brain axis. Biomedicines (2023) 11. doi: 10.3390/biomedicines11061701 PMC10296515 37371796

[12] Maes M Coucke F Leunis JC. NorMalization of the increased translocation of endotoxin from gram negative enterobacteria (leaky gut) is accompanied by a remission of chronic fatigue syndrome. Neuro Endocrinol Lett (2007) 28:739–44. 18063928

[13] Morris G Berk M Galecki P Maes M. The emerging role of autoimmunity in myalgic encephalomyelitis/chronic fatigue syndrome (ME/cfs). Mol Neurobiol (2014) 49:741–56. doi: 10.1007/s12035-013-8553-0 24068616

[14] Giloteaux L Goodrich JK Walters WA Levine SM Ley RE Hanson MR. Reduced diversity and altered composition of the gut microbiome in individuals with myalgic encephalomyelitis/chronic fatigue syndrome. Microbiome (2016) 4:30. doi: 10.1186/s40168-016-0171-4 27338587 PMC4918027

[15] Erdrich S Hawrelak JA Myers SP Harnett JE. Determining the association between fibromyalgia, the gut microbiome and its biomarkers: A systematic review. BMC Musculoskelet Disord (2020) 21:181. doi: 10.1186/s12891-020-03201-9 32192466 PMC7083062

[16] Goebel A Buhner S Schedel R Lochs H Sprotte G. Altered intestinal permeability in patients with primary fibromyalgia and in patients with complex regional pain syndrome. Rheumatol (Oxford) (2008) 47:1223–7. doi: 10.1093/rheumatology/ken140 18540025

[17] Othman M Aguero R Lin HC. Alterations in intestinal microbial flora and human disease. Curr Opin Gastroenterol (2008) 24:11–6. doi: 10.1097/MOG.0b013e3282f2b0d7 18043226

[18] Morris G Maes M Berk M Puri BK. Myalgic encephalomyelitis or chronic fatigue syndrome: how could the illness develop? Metab Brain Dis (2019) 34:385–415. doi: 10.1007/s11011-019-0388-6 30758706 PMC6428797

[19] Simeonova D Ivanovska M Murdjeva M Carvalho AF Maes M. Recognizing the leaky gut as a trans-diagnostic target for neuroimmune disorders using clinical chemistry and molecular immunology assays. Curr Top Med Chem (2018) 18:1641–55. doi: 10.2174/1568026618666181115100610 30430944

[20] Morris G Berk M Carvalho AF Caso JR Sanz Y Maes M. The role of microbiota and intestinal permeability in the pathophysiology of autoimmune and neuroimmune processes with an emphasis on inflammatory bowel disease type 1 diabetes and chronic fatigue syndrome. Curr Pharm Des (2016) 22:6058–75. doi: 10.2174/1381612822666160914182822 27634186

[21] Maes M Leunis JC. NorMalization of leaky gut in chronic fatigue syndrome (CFS) is accompanied by a clinical improvement: effects of age, duration of illness and the translocation of LPS from gram-negative bacteria. Neuro Endocrinol Lett (2008) 29:902–10. 19112401

[22] Maes M Mihaylova I Leunis JC. Increased serum IgA and IgM against LPS of enterobacteria in chronic fatigue syndrome (CFS): indication for the involvement of gram-negative enterobacteria in the etiology of CFS and for the presence of an increased gut-intestinal permeability. J Affect Disord (2007) 99:237–40. doi: 10.1016/j.jad.2006.08.021 17007934

[23] Shukla SK Cook D Meyer J Vernon SD Le T Clevidence D. Changes in gut and plasma microbiome following exercise challenge in myalgic encephalomyelitis/chronic fatigue syndrome (ME/CFS). PLoS One (2015) 10:e0145453. doi: 10.1371/journal.pone.0145453 26683192 PMC4684203

[24] Tuncer T Butun B Arman M Akyokus A Doseyen A. Primary fibromyalgia and allergy. Clin Rheumatol (1997) 16:9–12. doi: 10.1007/BF02238757 9132333

[25] Rowe PC Marden CL Jasion SE Cranston EM Flaherty MA Kelly KJ. Cow's milk protein intolerance in adolescents and young adults with chronic fatigue syndrome. Acta Paediatr (2016) 105:e412–8. doi: 10.1111/apa.13476 27177188

[26] Navaneetharaja N Griffiths V Wileman T Carding SR. A role for the intestinal microbiota and virome in myalgic encephalomyelitis/chronic fatigue syndrome (ME/CFS)? J Clin Med (2016) 5. doi: 10.3390/jcm5060055 PMC4929410 27275835

[27] Nagy-Szakal D Williams BL Mishra N Che X Lee B Bateman L. Fecal metagenomic profiles in subgroups of patients with myalgic encephalomyelitis/chronic fatigue syndrome. Microbiome (2017) 5:44. doi: 10.1186/s40168-017-0261-y 28441964 PMC5405467

[28] Nagy-Szakal D Barupal DK Lee B Che X Williams BL Kahn EJR. Insights into myalgic encephalomyelitis/chronic fatigue syndrome phenotypes through comprehensive metabolomics. Sci Rep (2018) 8:10056. doi: 10.1038/s41598-018-28477-9 29968805 PMC6030047

[29] Erdrich S Hawrelak JA Myers SP Vuyisich M Harnett JE. Investigating the association between the symptoms of women with Fibromyalgia, Digestive function, and markers of the microbiota of the Gastrointestinal Tract (The FIDGIT Study): study protocol. BMC Musculoskelet Disord (2023) 24:150. doi: 10.1186/s12891-023-06259-3 36849949 PMC9969038

[30] Wang XJ Ebbert JO Loftus CG Rosedahl JK Philpot LM. Comorbid extra-intestinal central sensitization conditions worsen irritable bowel syndrome in primary care patients. Neurogastroenterol Motil (2023) 35:e14546. doi: 10.1111/nmo.14546 36807964

[31] Berstad A Hauso O Berstad K Berstad JER. From IBS to ME - The dysbiotic march hypothesis. Med Hypotheses (2020) 140:109648. doi: 10.1016/j.mehy.2020.109648 32126475

[32] Cliff JM King EC Lee JS Sepulveda N Wolf AS Kingdon C. Cellular immune function in myalgic encephalomyelitis/chronic fatigue syndrome (ME/CFS). Front Immunol (2019) 10:796. doi: 10.3389/fimmu.2019.00796 31057538 PMC6477089

[33] Komaroff AL Bateman L. Will COVID-19 lead to myalgic encephalomyelitis/chronic fatigue syndrome? Front Med (Lausanne) (2020) 7:606824. doi: 10.3389/fmed.2020.606824 33537329 PMC7848220

[34] Armstrong CW McGregor NR Butt HL Gooley PR. Metabolism in chronic fatigue syndrome. Adv Clin Chem (2014) 66:121–72. doi: 10.1016/b978-0-12-801401-1.00005-0 25344988

[35] Guo C Che X Briese T Ranjan A Allicock O Yates RA. Deficient butyrate-producing capacity in the gut microbiome is associated with bacterial network disturbances and fatigue symptoms in ME/CFS. Cell Host Microbe (2023) 31:288–304 e8. doi: 10.1016/j.chom.2023.01.004 36758522 PMC10183837

[36] Malatji BG Meyer H Mason S Engelke UFH Wevers RA van Reenen M. A diagnostic biomarker profile for fibromyalgia syndrome based on an NMR metabolomics study of selected patients and controls. BMC Neurol (2017) 17:88. doi: 10.1186/s12883-017-0863-9 28490352 PMC5426044

[37] Malatji BG Mason S Mienie LJ Wevers RA Meyer H van Reenen M. The GC-MS metabolomics signature in patients with fibromyalgia syndrome directs to dysbiosis as an aspect contributing factor of FMS pathophysiology. Metabolomics (2019) 15:54. doi: 10.1007/s11306-019-1513-6 30919098

[38] Minerbi A Gonzalez E Brereton NJB Anjarkouchian A Dewar K Fitzcharles MA. Altered microbiome composition in individuals with fibromyalgia. Pain (2019) 160:2589–602. doi: 10.1097/j.pain.0000000000001640 31219947

[39] Minerbi A Fitzcharles MA. Gut microbiome: pertinence in fibromyalgia. Clin Exp Rheumatol (2020) 38 Suppl 123:99–104. 32116215

[40] Wolfe F Clauw DJ Fitzcharles MA Goldenberg DL Katz RS Mease P. The American College of Rheumatology preliminary diagnostic criteria for fibromyalgia and measurement of symptom severity. Arthritis Care Res (Hoboken) (2010) 62:600–10. doi: 10.1002/acr.20140 20461783

[41] Carruthers BM van de Sande MI De Meirleir KL Klimas NG Broderick G Mitchell T. Myalgic encephalomyelitis: international consensus criteria. J Intern Med (2011) 270:327–38. doi: 10.1111/j.1365-2796.2011.02428.x PMC3427890 21777306

[42] Fisk JD Ritvo PG Ross L Haase DA Marrie TJ Schlech WF. Measuring the functional impact of fatigue: initial validation of the fatigue impact scale. Clin Infect Dis (1994) 18 Suppl 1:S79–83. doi: 10.1093/clinids/18.supplement_1.s79 8148458

[43] Sletten DM Suarez GA Low PA Mandrekar J Singer W. COMPASS 31: a refined and abbreviated Composite Autonomic Symptom Score. Mayo Clin Proc (2012) 87:1196–201. doi: 10.1016/j.mayocp.2012.10.013 PMC3541923 23218087

[44] Buysse DJ Reynolds CF 3rd Monk TH Berman SR Kupfer DJ. The Pittsburgh Sleep Quality Index: a new instrument for psychiatric practice and research. Psychiatry Res (1989) 28:193–213. doi: 10.1016/0165-1781(89)90047-4 2748771

[45] Alonso J Prieto L Anto JM. [The Spanish version of the SF-36 Health Survey (the SF-36 health questionnaire): an instrument for measuring clinical results]. Med Clin (Barc) (1995) 104:771–6. 7783470

[46] Morcillo S Atencia JA Martín F Ortega A Bilbao JR Rubio-Martín E. Consumption of cows’ milk is associated with lower risk of type 2 diabetes mellitus. A cross-sectional study Int Dairy J (2012) 26:162–65. doi: 10.1016/j.idairyj.2012.03.011

[47] Maes M Andres-Rodriguez L Vojdani A Sirivichayakul S Barbosa DS Kanchanatawan B. In schizophrenia, chronic fatigue syndrome- and fibromyalgia-like symptoms are driven by breakdown of the paracellular pathway with increased zonulin and immune activation-associated neurotoxicity. CNS Neurol Disord Drug Targets (2023) 22:215–25. doi: 10.2174/1871527321666220806100600 35946099

[48] Safadi JM Quinton AMG Lennox BR Burnet PWJ Minichino A. Gut dysbiosis in severe mental illness and chronic fatigue: a novel trans-diagnostic construct? A systematic review and meta-analysis. Mol Psychiatry (2022) 27:141–53. doi: 10.1038/s41380-021-01032-1 PMC8960409 33558650

[49] De Meirleir KL Mijatovic T Subramanian K Schlauch KA Lombardi VC. Evaluation of four clinical laboratory parameters for the diagnosis of myalgic encephalomyelitis. J Transl Med (2018) 16:322. doi: 10.1186/s12967-018-1696-z 30463572 PMC6249861

[50] Uhde M Indart AC Green PHR Yolken RH Cook DB Shukla SK. Suppressed immune and metabolic responses to intestinal damage-associated microbial translocation in myalgic encephalomyelitis/chronic fatigue syndrome. Brain Behav Immun Health (2023) 30:100627. doi: 10.1016/j.bbih.2023.100627 37396339 PMC10308215

[51] Lakhan SE Kirchgessner A. Gut inflammation in chronic fatigue syndrome. Nutr Metab (Lond) (2010) 7:79. doi: 10.1186/1743-7075-7-79 20939923 PMC2964729

[52] Maes M Bosmans E Kubera M. Increased expression of activation antigens on CD8+ T lymphocytes in Myalgic Encephalomyelitis/chronic fatigue syndrome: inverse associations with lowered CD19+ expression and CD4+/CD8+ ratio, but no associations with (auto)immune, leaky gut, oxidative and nitrosative stress biomarkers. Neuro Endocrinol Lett (2015) 36:439–46. 26707044

[53] Sheedy JR Wettenhall RE Scanlon D Gooley PR Lewis DP McGregor N. Increased d-lactic Acid intestinal bacteria in patients with chronic fatigue syndrome. In Vivo (2009) 23:621–8. 19567398

[54] Valencia C Fatima H Nwankwo I Anam M Maharjan S Amjad Z. A correlation between the pathogenic processes of fibromyalgia and irritable bowel syndrome in the middle-aged population: A systematic review. Cureus (2022) 14:e29923. doi: 10.7759/cureus.29923 36381861 PMC9635936

[55] De Meirleir KL Khaiboullina SF Fremont M Hulstaert J Rizvanov AA Palotas A. Plasmacytoid dendritic cells in the duodenum of individuals diagnosed with myalgic encephalomyelitis are uniquely immunoreactive to antibodies to human endogenous retroviral proteins. In Vivo (2013) 27:177–87. PMC3776582 23422476

[56] Pimentel M Wallace D Hallegua D Chow E Kong Y Park S. A link between irritable bowel syndrome and fibromyalgia may be related to findings on lactulose breath testing. Ann Rheum Dis (2004) 63:450–2. doi: 10.1136/ard.2003.011502 PMC1754959 15020342

