# Supplementary figures and images for: Increased gut permeability and bacterial translocation are associated with fibromyalgia and myalgic encephalomyelitis/chronic fatigue syndrome: implications for disease-related biomarker discovery
Source: Front Immunol. 2023 Sep 7;14:1253121. doi: 10.3389/fimmu.2023.1253121 (PMC10512706; doi:10.3389/fimmu.2023.1253121)

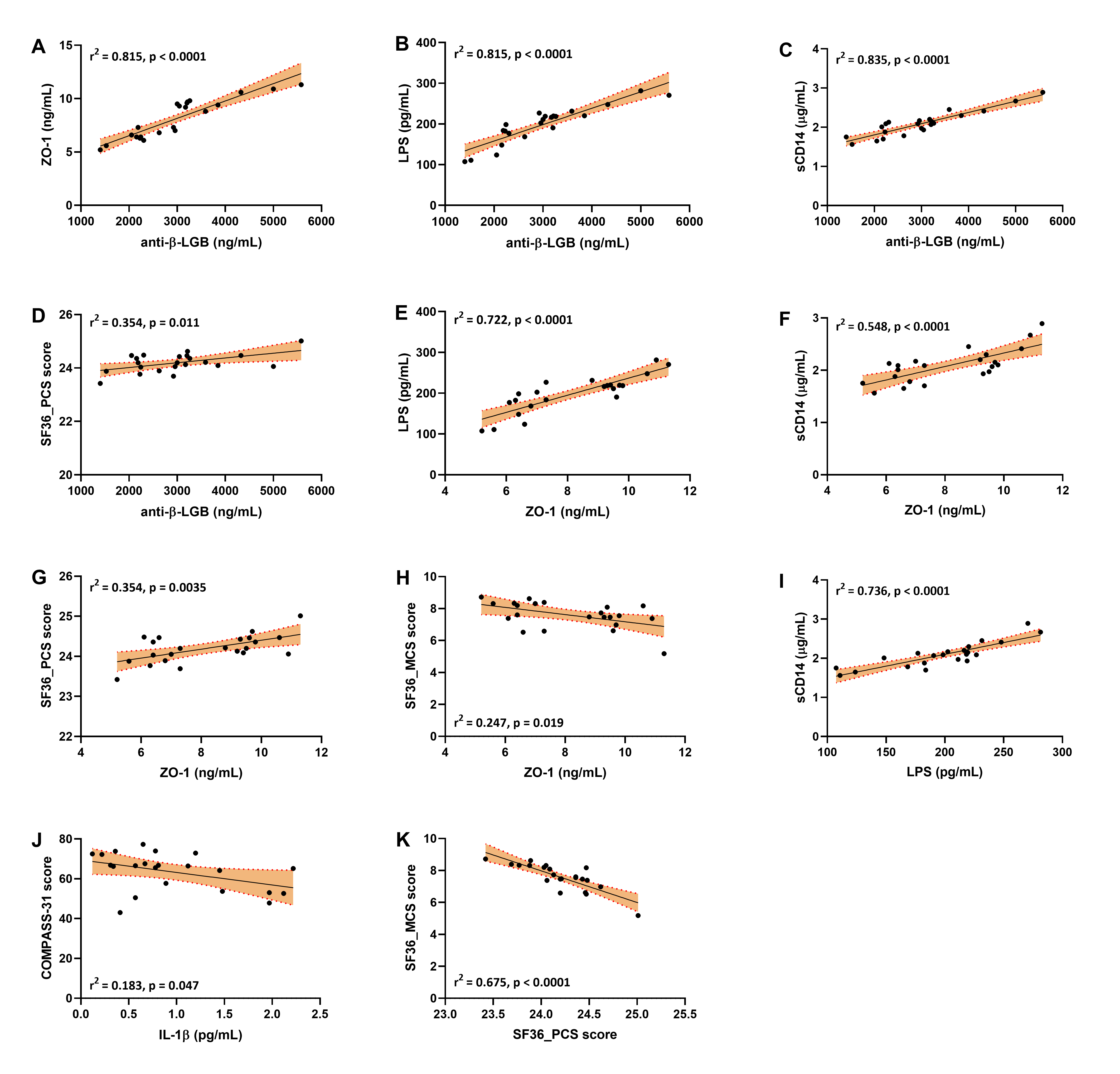

Supplement: Supplementary Figure 1 — Multipanel scatter dot plots depicting the statistically significant between intestinal barrier function biomarkers and self-reported outcome measures in fibromyalgia patients. Each dot corresponds to an individual. Spearman’s correlation scatter plots with linear regression (black line) and the 95% confidence interval (brown band) was used to calculate the association. Square Spearman’s rank correlation coefficient (rho2) and statistically significant p-values are shown in each panel. [file Image_1.tif]

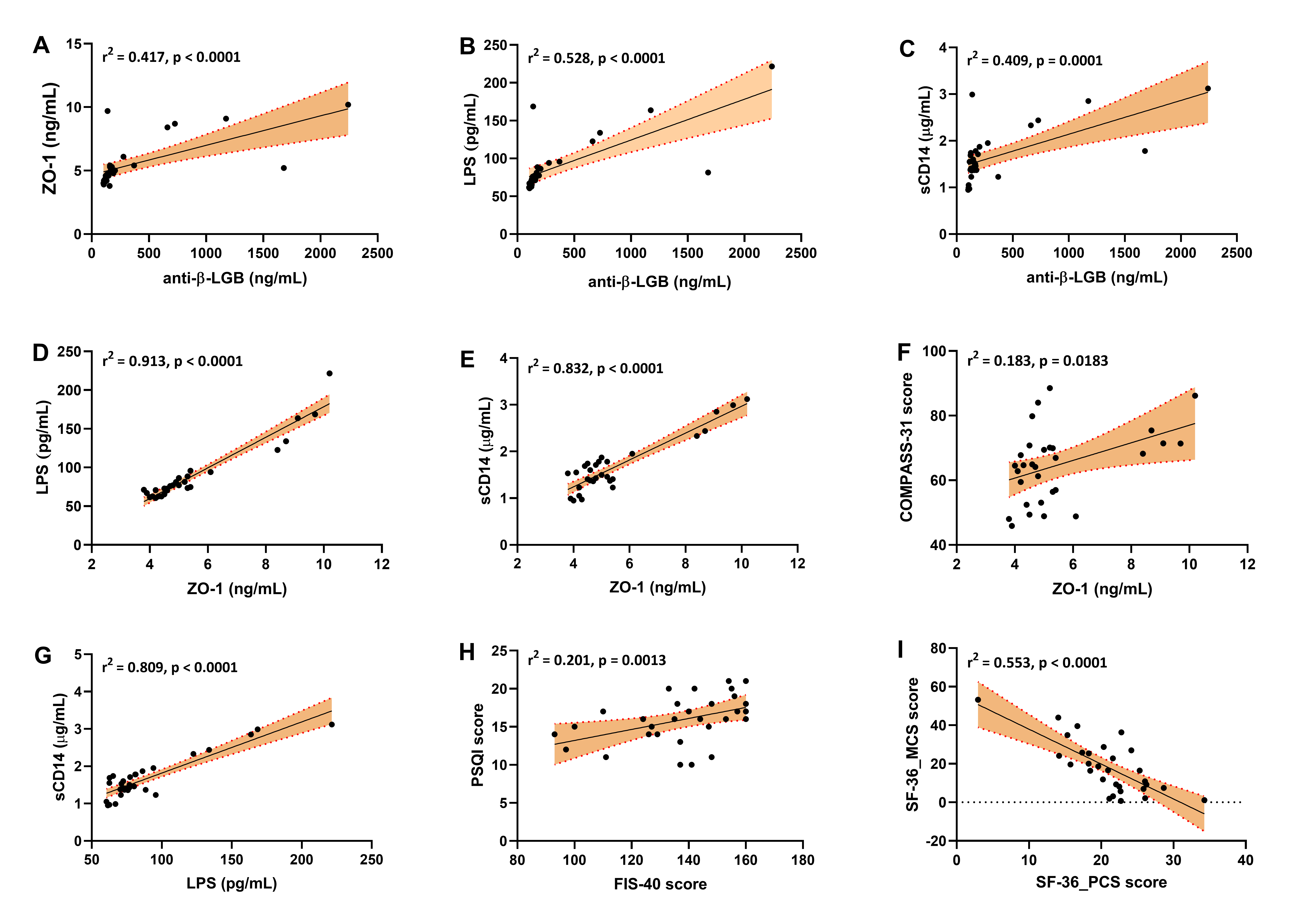

Supplement: Supplementary Figure 2 — Multipanel scatter dot plots depicting the statistically significant between intestinal barrier function biomarkers and self-reported outcome measures in ME/CFS patients. Each dot corresponds to an individual. Spearman’s correlation scatter plots with linear regression (black line) and the 95% confidence interval (brown band) was used to calculate the association. Square Spearman’s rank correlation coefficient (rho2) and statistically significant p-values are shown in each panel. [file Image_2.tif]

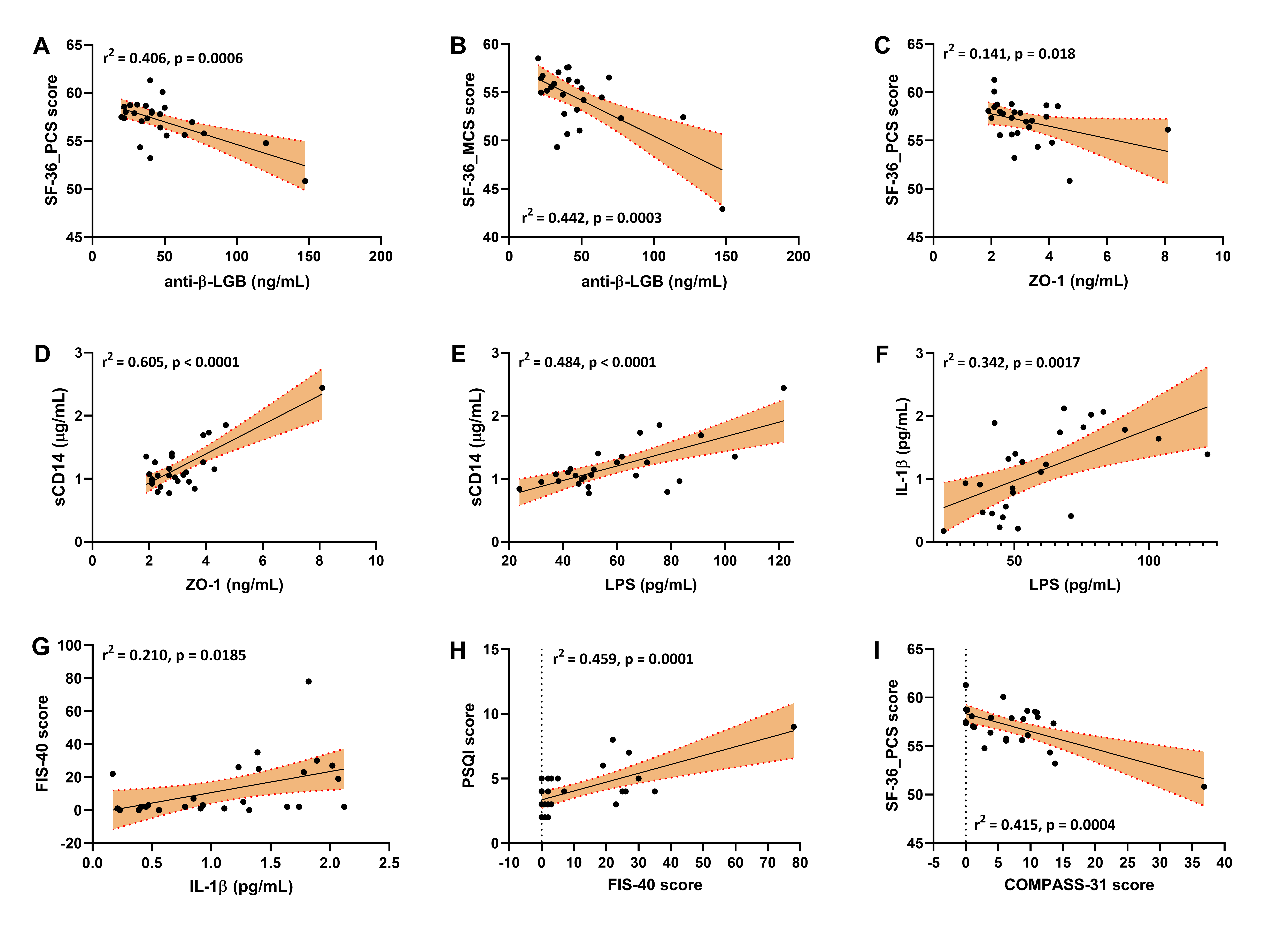

Supplement: Supplementary Figure 3 — Multipanel scatter dot plots depicting the statistically significant between intestinal barrier function biomarkers and self-reported outcome measures in healthy controls. Each dot corresponds to an individual. Spearman’s correlation scatter plots with linear regression (black line) and the 95% confidence interval (brown band) was used to calculate the association. Square Spearman’s rank correlation coefficient (rho2) and statistically significant p-values are shown in each panel. [file Image_3.tif]
